# Crystal structures of three substituted 3-aryl-2-phenyl-2,3-di­hydro-4*H*-1,3-benzo­thia­zin-4-ones

**DOI:** 10.1107/S2056989016011002

**Published:** 2016-07-12

**Authors:** Hemant P. Yennawar, David J. Coyle, Duncan J. Noble, Ziwei Yang, Lee J. Silverberg

**Affiliations:** a Department of Chemistry, Pennsylvania State University, University Park, PA 16802, USA; b Pennsylvania State University, Schuylkill Campus, 200 University Drive, Schuylkill Haven, PA 17972, USA

**Keywords:** crystal structure, benzo­thia­zinones, screw-boat thia­zine pucker, aromatic ring inter­actions

## Abstract

Three ring-substituted 3-aryl analogs of 2-phenyl-2,3-di­hydro-4*H*-1,3-benzo­thia­zin-4-one, namely 3-(4-meth­oxy­phen­yl)-2-phenyl-4*H*-1,3-benzo­thia­zin-4-one, C_21_H_17_NO_2_S, (I), 2-phenyl-3-[4-(tri­fluoro­meth­yl)phen­yl]-2,3-di­hydro-4*H*-1,3-benzo­thia­zin-4-one toluene hemisolvate, C_21_H_14_F_3_NOS·0.5C_7_H_8_, (II), and 3-(3-bromo­phen­yl)-2-phenyl-2,3-di­hydro-4*H*-1,3-benzo­thia­zin-4-one toluene hemisolvate, C_20_H_14_BrNOS·0.5C_7_H_8_, (III), were synthesized and their crystal structures determined. The hemisolvates differ in that in (II), the asymmetric unit comprises two molecules of the benzo­thia­zinone compound and a toluene solvent mol­ecule, whereas in (III), the unit comprises one benzo­thia­zinone mol­ecule and a half-occupancy toluene solvent mol­ecule. All crystals are of racemic mixtures of the chiral 2-C atom of the thia­zine moiety, which in all structures has a screw-boat puckering, with the puckering amplitude values within the range 0.575–0.603 Å. In all three structures, the benzene plane of the benzo­thia­zine system makes a dihedral angle in the range 78.60 (5) to 98.40 (5)° with the unsubstituted benzene plane and in the range 70.50 (1) to 121.00 (5)° with the substituted benzene plane. The CF_3_ substituent group in one of the mol­ecules of (II) shows positional disorder, with an occupancy ratio of 0.57 (3):0.43 (3). In the crystals of (I) and (II), weak inter­molecular C—H⋯O inter­actions are present, giving in (I), mol­ecules arranged in a plane parallel to (010), and in (II), chains along *a*. In addition, all three structures show weak C—H⋯π inter­actions involving various aromatic rings.

## Abstract 2

The crystal structures of three closely related benzo­thia­zinone structures are reported. All three are conformationally similar having screw-boat puckering in the thia­zine ring and C—H⋯π-type inter­molecular inter­actions in the crystals.

## Chemical context   

We have previously reported the crystal structures of 2,3-diphenyl-2,3-di­hydro-4*H*-1,3-benzo­thia­zin-4-one (Yennawar *et al.*, 2014 ▸) and three 2-aryl-3-phenyl-2,3-di­hydro-4*H*-1,3-benzo­­thia­zin-4-ones (Yennawar *et al.*, 2013 ▸, 2015 ▸). In the pre­sent communication, we report the synthesis and crystal structures of three ring-substituted 3-aryl-2-phenyl-2,3-di­hydro-4*H*-1,3-benzo­thia­zin-4-ones, namely the 4-meth­oxy­phenyl com­pound, (I), the 4-(tri­fluoro­meth­yl)phenyl com­pound as the toluene hemisolvate, (II), and the 4-bromo­phenyl compound as the toluene hemisolvate, (III). However, (II) and (III) differ in that the asymmetric unit of (II) comprises two independent benzo­thia­zinone mol­ecules and one toluene solvent mol­ecule, while that of (III) comprises one benzo­thia­zinone mol­ecule and a half-occupancy toluene solvent mol­ecule. Each compound has been synthesized using the same T3P/pyridine (T3P is 2,4,6-tripropyl-1,3,5,2,4,6-trioxatriphosphorinane 2,4,6-trioxide) method that was used for the preparation of the previously mentioned analogous compounds (Yennawar *et al.*, 2013 ▸, 2014 ▸, 2015 ▸).
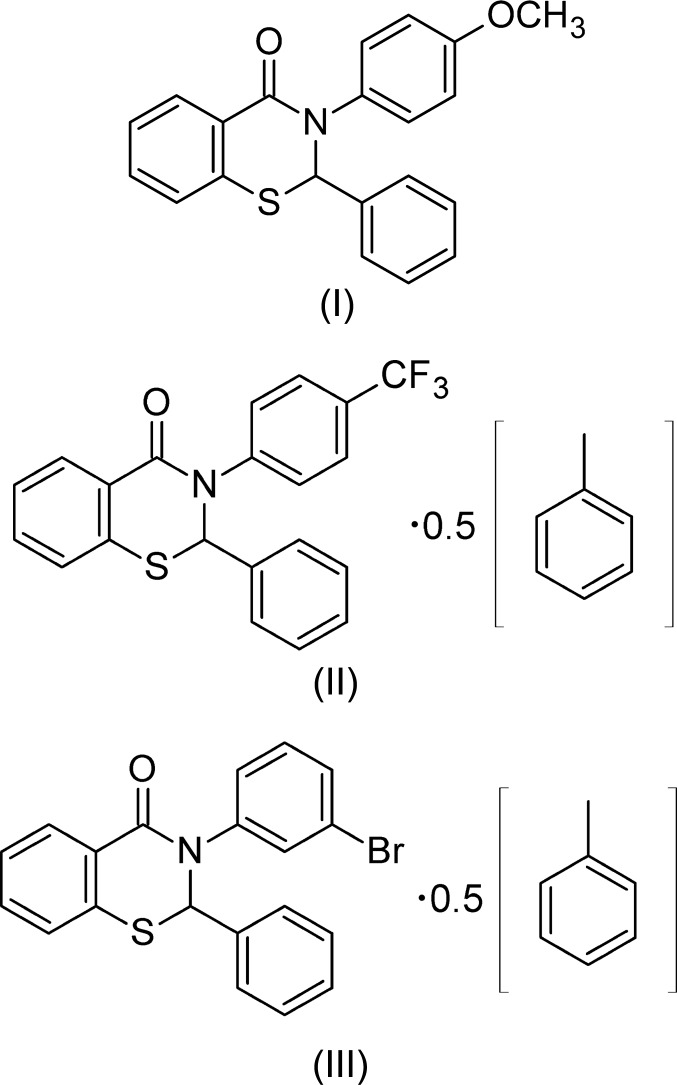


## Structural commentary   

**Figure 1:**
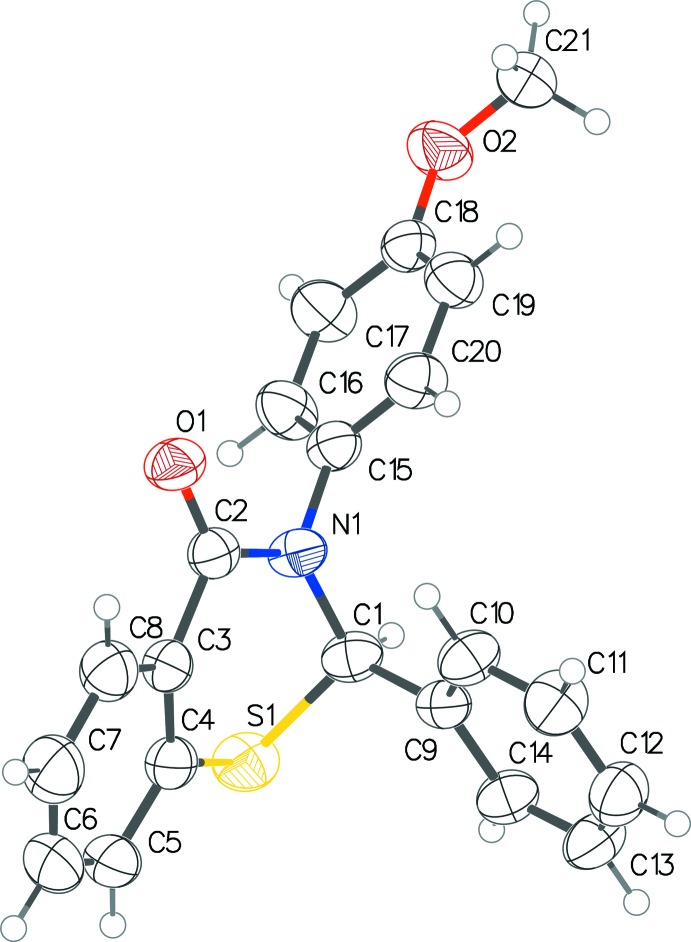
The mol­ecular conformation and atom-numbering scheme for (I), with non-H atoms shown as 50% probability displacement ellipsoids.

The three benzo­thia­zinones (Figs. 1 ▸–3 ▸ ▸) exhibit fairly similar conformations. In all three, the thia­zine ring pucker is screw-boat, with θ between 63.0 and 67.1°, and puckering amplitudes within the range 0.575–0.603 Å. The inter­planar angle between the benzene ring of the benzo­thia­zine system and the substituent benzene rings at the 2-position are 82.68 (6)° in (I), 95.69 (5) and 78.10 (5)° in (II), and 98.37 (1)° in (III). Those with the benzene rings at the 3-position are 59.10 (6)° in (I), 70.56 (5) and 72.26 (5)° in (II), and 78.66 (1)° in (III). The CF_3_ substituent group in one of the mol­ecules of (II) shows positional disorder, with an occupancy ratio of 0.57 (3):0.43 (3).

## Supra­molecular features   

**Figure 4:**
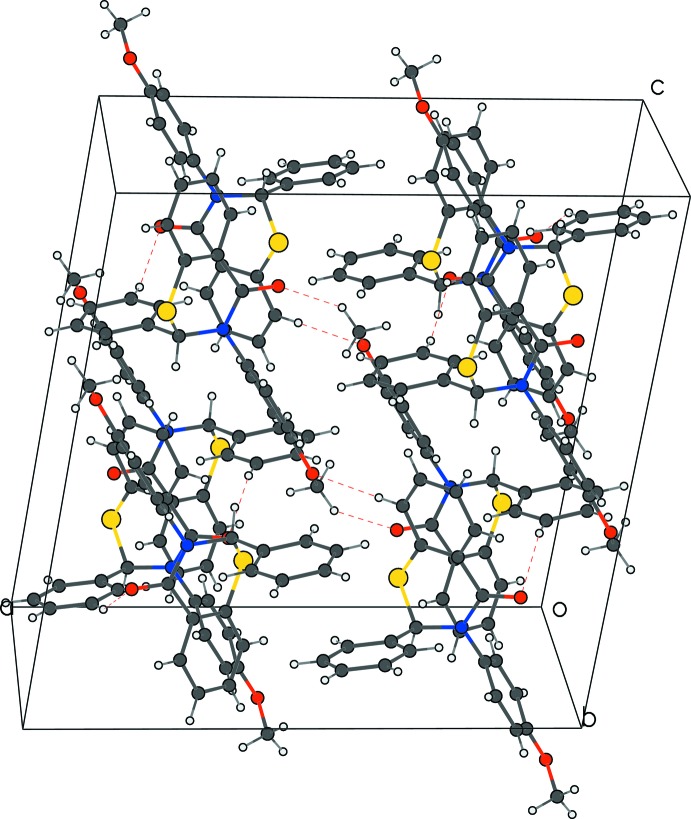
The crystal packing of (I) in the unit cell, viewed along *b*, showing C—H⋯O hydrogen bonds as dashed lines.

**Figure 5:**
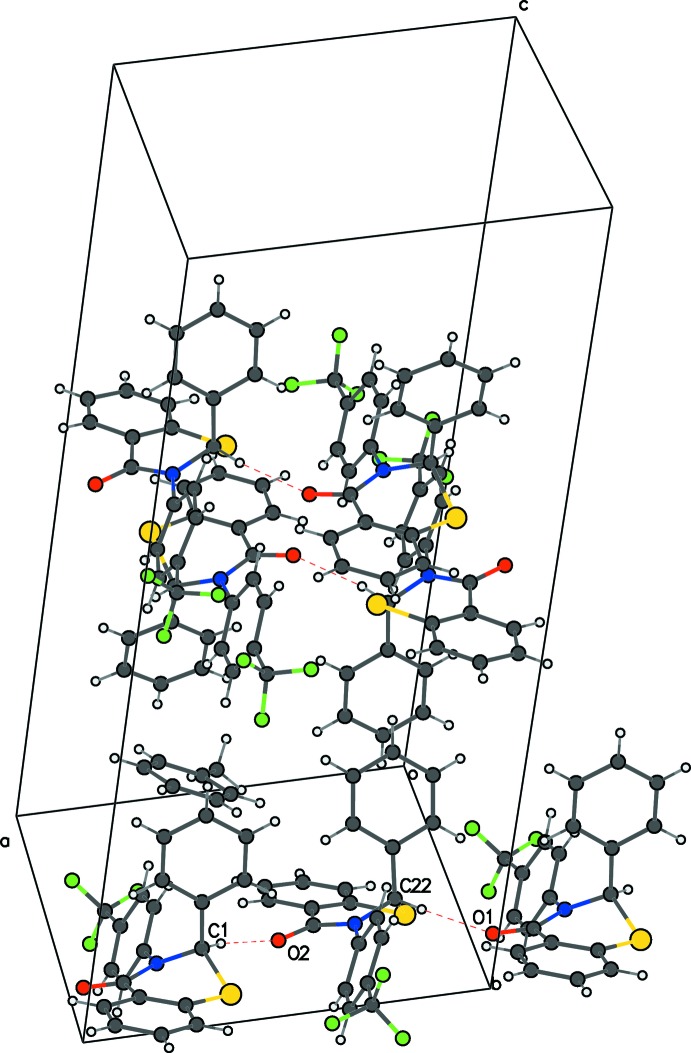
The crystal packing of (II) in the unit cell, viewed along *b*, showing C—H⋯O hydrogen bonds as dashed lines.

**Figure 6:**
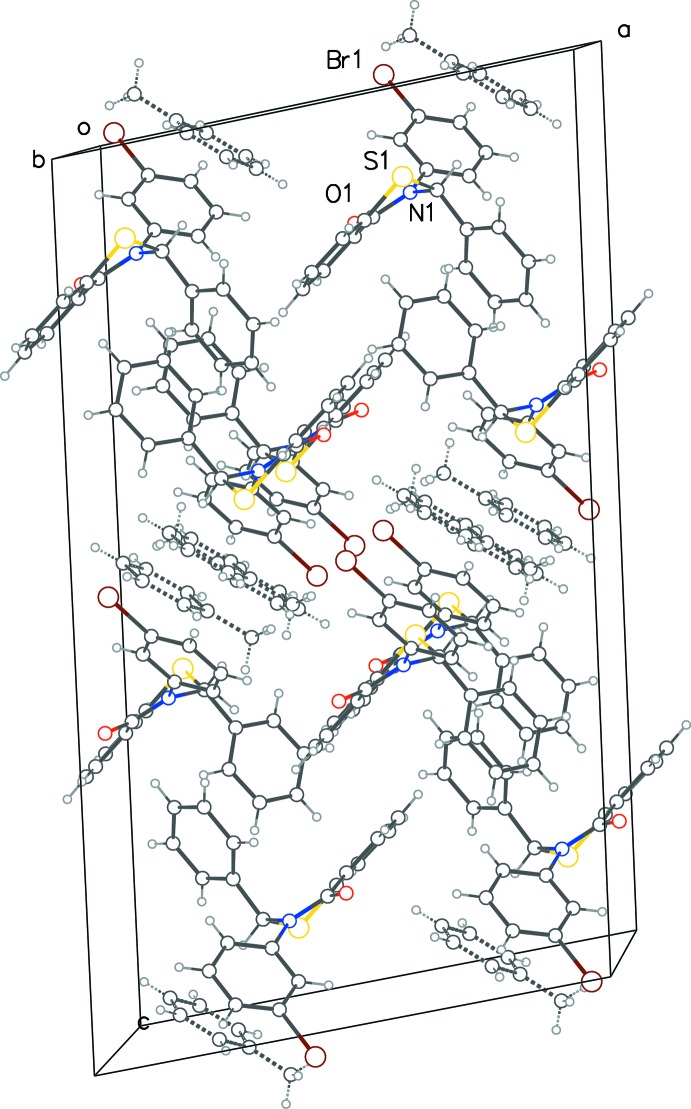
A perspective view of the crystal packing of (III), with the half-occupancy toluene solvent mol­ecules shown as dashed bonds.

In (I) and (II), weak inter­molecular C—H⋯O inter­actions are observed (Tables 1 ▸ and 2 ▸, respectively), giving in (I), mol­ecules arranged in a plane parallel to (010) (Fig. 4 ▸), and in (II), chains along the *a*-axis direction (Fig. 5 ▸). The crystals also feature T-type C—H⋯π inter­actions (Tables 1 ▸–3 ▸ ▸), as analyzed using *PLATON* (Spek, 2009 ▸). In (I), a weak C—H⋯*Cg*(π ring) inter­action of 3.8068 (10) Å is present with an inter­acting angle of 148°. In (II), the toluene mol­ecule participates in tilted-T-type inter­actions by placing itself obliquely between phenyl rings of the two enanti­omers, with C—H⋯*Cg*(toluene) distances of 3.5916 (7) and 3.6009 (7) Å, with inter­acting angles of 145 and 147°, respectively. In (III), two C—H⋯π inter­actions, one between the thia­zine ring and the toluene solvent mol­ecule and the other between the fused benzene ring and the 2-phenyl ring, have C—H⋯*Cg* distances of 3.5802 (6) and 3.6823 (6) Å, with inter­acting angles of 156 and 153°, respectively (Fig. 6 ▸). Structure (I) also shows a very weak parallel-displaced π–π inter­action between symmetry-related benzene rings, with an inter-centroid (*Cg⋯*Cg**) distance of 3.977 (1) Å and an inter­planar angle of 8°.

## Database survey   

The three structures reported here and four previously reported analogous structures (Yennawar *et al.*, 2013 ▸, 2014 ▸, 2015 ▸) have very similar screw-boat puckering for the thia­zine ring. Among the seven crystal structures, the variation in the inter­planar angles between the benzene ring of the benzo­thia­zine moiety and the two substituent benzene rings at positions 2 and 3 lie within 26 and 30°, respectively. A structure for 2-(5-methyl­thio­phen-2-yl)-3-phenyl-2,3-di­hydro­quin­az­olin-4(1*H*)-one has been reported in a patent application (Atwood *et al.*, 2015 ▸).

## Synthesis and crystallization   

A two-necked 25 ml round-bottomed flask was oven-dried, cooled under N_2_ and charged with a stir bar and an *N*-aryl-*C*-phenylimine (6 mmol). Tetra­hydro­furan or 2-methyl­tetra­hydro­furan (2.3 ml) was added, the solid dissolved and the solution stirred. Pyridine (1.95 ml, 24 mmol) was added, followed by thio­salicylic acid (0.93 g, 6 mmol). Finally, 2,4,6-tri­propyl-1,3,5,2,4,6-trioxatri­phospho­rinane 2,4,6-tri­ox­ide (T3P) in 2-methyl­tetra­hydro­furan (50 wt%, 7.3 ml, 12 mmol) was added. The mixture was stirred at room temperature and the reaction was followed using thin-layer chromatography. The mixture was then poured into a separatory funnel and di­chloro­methane and distilled water were added. The layers were separated and the aqueous layer was then extracted twice with di­chloro­methane. The organics were combined and washed with saturated sodium bicarbonate and then saturated sodium chloride. The organic extract was dried over sodium sulfate and concentrated under vacuum. The crude product was chromatographed on 30 g of flash silica gel using mixtures of ethyl acetate and hexa­nes, and then further purified as indicated below.

Compound (I) was recrystallized from ethanol solution to give yellow crystals (yield 0.72 g, 34.6%; m.p. 365–369 K). *R*_F_ = 0.52 (50% ethyl acetate/hexa­nes). Colorless block-shaped crystals suitable for the X-ray analysis were grown by slow evaporation from ethanol solution.

Compound (II) was recrystallized from methyl­ene chloride/hexa­nes to give yellow crystals (yield 0.5639 g, 24.4%; m.p. 404–406 K). *R*_F_ = 0.56 (30% ethyl acetate/hexa­nes solution). Colorless needle-shaped crystals suitable for the X-ray analysis were grown by slow evaporation from toluene solution.

Compound (III) was triturated with hexa­nes solution to give a solid (0.7242 g) and then recrystallized from toluene/hexa­nes to give white crystals (yield 0.3544 g, 14.5%; m.p.: 358–359 K). *R*_F_ = 0.39 (20% ethyl acetate/hexa­nes). A second crop of 0.30 g (12.7%) was obtained by slow evaporation of the mother liquor, giving colorless blocks suitable for the X-ray analysis.

## Refinement   

Crystal data, data collection and structure refinement details are summarized in Table 4 ▸. In the refinement of (II), the two mol­ecules in the asymmetric unit were restrained using the SAME command in *SHELXL2014* (Sheldrick, 2015 ▸). One of the mol­ecules shows positional disorder in the –CF_3_ group, with the occupancy ratio refining to 0.57 (3):0.43 (3). We tried to address the high *R* values (relative to *R*_int_) by looking for twinning and using restraints but we have had no success in achieving respectable *R* values. In (III), the disordered partial toluene mol­ecule was refined with a site-occupancy factor determined as 0.50 and with positional constraints (AFIX 6). In all three compounds, the H atoms were placed geometrically and allowed to ride on the C atoms during refinement, with C—H distances of 0.98 (methine), 0.96 (meth­yl) or 0.93 Å (aromatic) and with *U*_iso_(H) = 1.5*U*_eq_(C) for methyl H atoms and 1.2*U*_eq_(C) otherwise.

## Supplementary Material

Crystal structure: contains datablock(s) 1, II, III, I. DOI: 10.1107/S2056989016011002/zs2362sup1.cif

Supporting information file. DOI: 10.1107/S2056989016011002/zs2362Isup2.cml

Supporting information file. DOI: 10.1107/S2056989016011002/zs2362IIsup3.cml

Supporting information file. DOI: 10.1107/S2056989016011002/zs2362IIIsup4.cml

CCDC references: 1491202, 1491201, 1491200

Additional supporting information: crystallographic information; 3D view; checkCIF report

## Figures and Tables

**Figure 2:**
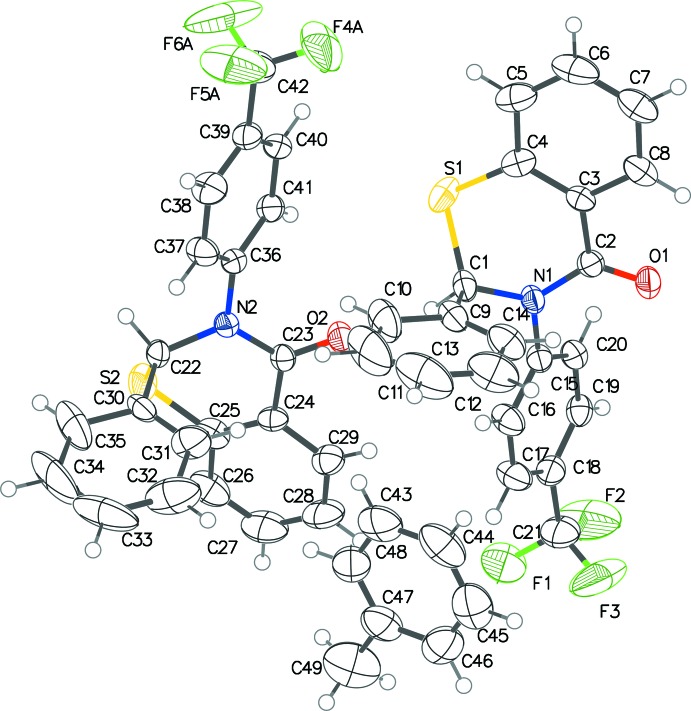
The mol­ecular conformation and atom-numbering scheme for (II), with non-H atoms shown as 50% probability displacement ellipsoids. The minor component of the disordered CF_3_ group is not shown.

**Figure 3:**
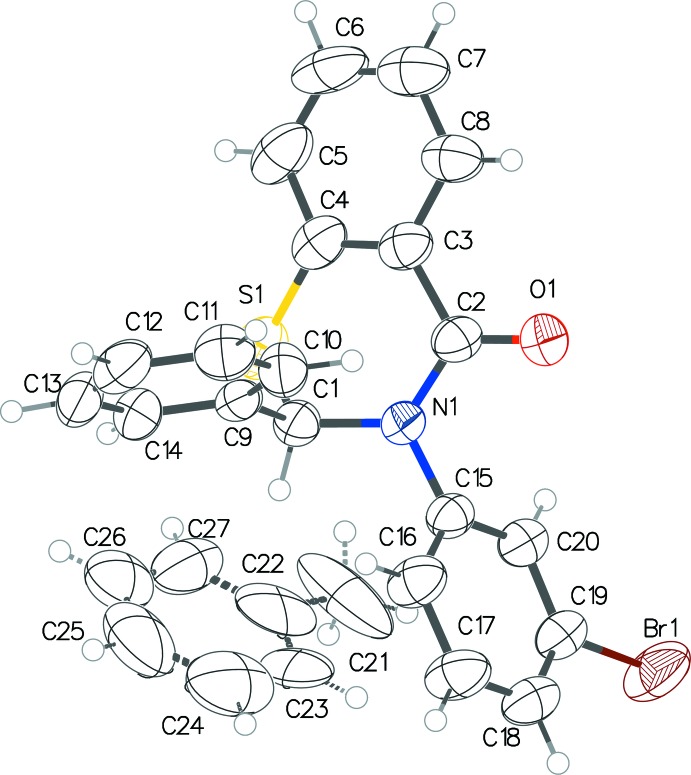
The mol­ecular conformation and atom-numbering scheme for (III), with non-H atoms shown as 50% probability ellipsoids. The partial-occupancy disordered toluene solvent mol­ecule has a site occupancy of 0.50.

**Table 1:** Hydrogen-bond geometry (Å, °) for (I) *CgX* = center of gravity of ring *X*; *D*—H⋯*CgX* = angle of the *D*—H bond with the π plane

*D*—H⋯*A*	*D*—H	H⋯*A*	*D*⋯*A*	*D*—H⋯*A*
C11—H11⋯O1^i^	0.93	2.59	3.447 (2)	154
C5—H5⋯O2^ii^	0.93	2.46	3.387 (2)	173
C21—H21*A*⋯*Cg*4^iii^	0.96	2.96	3.8068 (10)	148

Symmetry codes: (i) 
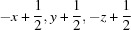
; (ii) 
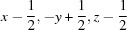
; (iii) 
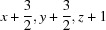
.

**Table 2:** Hydrogen-bond geometry (Å, °) for (II) *Cg* *X* = center of gravity of ring *X*; *D*—H⋯*CgX* = angle of the *D*—H bond with the π plane

*D*—H⋯*A*	*D*—H	H⋯*A*	*D*⋯*A*	*D*—H⋯*A*
C1—H1⋯O2	0.98	2.25	3.2053 (7)	165
C22—H22⋯O1^i^	0.98	2.34	3.3140 (7)	171
C17—H17⋯*Cg*9	0.93	2.79	3.5916 (7)	145
C38—H38⋯*Cg*9^ii^	0.93	2.78	3.6009 (7)	147

Symmetry codes: (i) 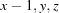; (ii) 
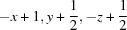
.

**Table 3:** Hydrogen-bond geometry (Å, °) for (III) *CgX* = center of gravity of ring *X*; *D*—H⋯*CgX* = angle of the *D*—H bond with the π plane

*D*—H⋯*A*	*D*—H	H⋯*A*	*D*⋯*A*	*D*—H⋯*A*
C1—H1⋯*Cg*5	0.98	2.66	3.5802 (6)	156
C6—H6⋯*Cg*3^i^	0.93	2.83	3.6823 (6)	153

Symmetry code: (i) 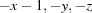.

**Table 4:** Experimental details

	(I)	(II)	(III)
Crystal data			
Chemical formula	C_21_H_17_NO_2_S	2C_21_H_14_F_3_NOS·C_7_H_8_	2C_20_H_14_BrNOS·C_7_H_8_
*M*_r_	347.42	862.92	884.72
Crystal system, space group	Monoclinic, *C*2/*c*	Monoclinic, *P*2_1_/*c*	Monoclinic, *C*2/*c*
Temperature (K)	298	298	298
*a*, *b*, *c* (Å)	17.820 (4), 11.016 (3), 17.890 (4)	11.953 (2), 14.516 (3), 24.546 (5)	15.736 (2), 9.3530 (15), 27.259 (4)
β (°)	98.385 (5)	101.024 (4)	99.560 (3)
*V* (Å^3^)	3474.4 (15)	4180.5 (14)	3956.2 (10)
*Z*	8	4	4
Radiation type	Mo *K*α	Mo *K*α	Mo *K*α
μ (mm^−1^)	0.20	0.20	2.20
Crystal size (mm)	0.26 × 0.24 × 0.12	0.29 × 0.09 × 0.07	0.21 × 0.17 × 0.10

Data collection			
Diffractometer	Bruker CCD area-detector	Bruker CCD area-detector	Bruker CCD area-detector
Absorption correction	Multi-scan (*SADABS*; Bruker, 2001 ▸)	Multi-scan (*SADABS*; Bruker, 2001 ▸)	Multi-scan (*SADABS*; Bruker, 2001 ▸)
*T*_min_, *T*_max_	0.940, 0.986	0.592, 0.920	0.103, 0.901
No. of measured, independent and observed reflections	15005, 4284, 3414 [*I* > 2σ(*I*)]	39335, 10359, 8329 [*I* > 2σ(*I*)]	18289, 4903, 2424 [*I* > 2σ(*I*)]
*R*_int_	0.024	0.052	0.064
(sin θ/λ)_max_ (Å^−1^)	0.666	0.668	0.667

Refinement			
*R*[*F* ^2^ > 2σ(*F* ^2^)], *wR*(*F* ^2^), *S*	0.045, 0.125, 1.05	0.133, 0.240, 1.31	0.049, 0.153, 0.79
No. of reflections	4284	10359	4903
No. of parameters	227	580	266
No. of restraints	0	74	186
H-atom treatment	H-atom parameters constrained	H-atom parameters constrained	H-atom parameters constrained
Δρ_max_, Δρ_min_ (e Å^−3^)	0.22, −0.37	0.48, −0.34	0.73, −0.71

Computer programs: *SMART* and *SAINT* (Bruker, 2001 ▸), *SHELXS97* and *SHELXL97* (Sheldrick, 2008 ▸), *SHELXL2014* (Sheldrick, 2015 ▸) and *OLEX2* (Dolomanov *et al.*, 2009 ▸).

## supplementary crystallographic information

### Crystal data

**Table:**

2C_20_H_14_BrNOS·C_7_H_8_	*D*_x_ = 1.485 Mg m^−^^3^
*M**_r_* = 884.72	Melting point = 358–359 K
Monoclinic, *C*2/*c*	Mo *K*α radiation, λ = 0.71073 Å
*a* = 15.736 (2) Å	Cell parameters from 3326 reflections
*b* = 9.3530 (15) Å	θ = 2.5–25.0°
*c* = 27.259 (4) Å	µ = 2.20 mm^−^^1^
β = 99.560 (3)°	*T* = 298 K
*V* = 3956.2 (10) Å^3^	Block, colorless
*Z* = 4	0.21 × 0.17 × 0.10 mm
*F*(000) = 1800	

### Data collection

**Table:**

Bruker CCD area-detector diffractometer	2424 reflections with *I* > 2σ(*I*)
phi and ω scans	*R*_int_ = 0.064
Absorption correction: multi-scan (SADABS; Bruker, 2001)	θ_max_ = 28.3°, θ_min_ = 2.5°
*T*_min_ = 0.103, *T*_max_ = 0.901	*h* = −20→20
18289 measured reflections	*k* = −12→12
4903 independent reflections	*l* = −36→36

### Refinement

**Table:**

Refinement on *F*^2^	Primary atom site location: structure-invariant direct methods
Least-squares matrix: full	Hydrogen site location: mixed
*R*[*F*^2^ > 2σ(*F*^2^)] = 0.049	H-atom parameters constrained
*wR*(*F*^2^) = 0.153	*w* = 1/[σ^2^(*F*_o_^2^) + (0.1*P*)^2^] where *P* = (*F*_o_^2^ + 2*F*_c_^2^)/3
*S* = 0.79	(Δ/σ)_max_ < 0.001
4903 reflections	Δρ_max_ = 0.73 e Å^−^^3^
266 parameters	Δρ_min_ = −0.71 e Å^−^^3^
186 restraints	

### Special details

**Table:**

Experimental. Absorption correction: SADABS was used for absorption correction. R(int) was 0.2680 before and 0.0355 after correction. The Ratio of minimum to maximum transmission is 0.1027. The λ/2 correction factor is 0.0015. The data collection nominally covered a full sphere of reciprocal space by a combination of 4 sets of ω scans each set at different φ and/or 2θ angles and each scan (10 s exposure) covering -0.300° degrees in ω. The crystal to detector distance was 5.82 cm.
Geometry. All esds (except the esd in the dihedral angle between two l.s. planes) are estimated using the full covariance matrix. The cell esds are taken into account individually in the estimation of esds in distances, angles and torsion angles; correlations between esds in cell parameters are only used when they are defined by crystal symmetry. An approximate (isotropic) treatment of cell esds is used for estimating esds involving l.s. planes.

### Fractional atomic coordinates and isotropic or equivalent isotropic displacement parameters (Å^2^)

**Table:**

	*x*	*y*	*z*	*U*_iso_*/*U*_eq_	Occ. (<1)
Br1	0.45074 (3)	0.27453 (5)	0.01191 (2)	0.0874 (2)	
C1	0.3189 (2)	−0.2500 (3)	−0.12554 (12)	0.0471 (8)	
H1	0.2798	−0.2262	−0.1023	0.057*	
C2	0.4549 (2)	−0.1391 (3)	−0.14162 (11)	0.0479 (8)	
C3	0.4821 (2)	−0.2827 (3)	−0.15744 (12)	0.0506 (8)	
C4	0.4518 (2)	−0.4113 (3)	−0.14088 (11)	0.0540 (9)	
C5	0.4858 (3)	−0.5405 (4)	−0.15463 (15)	0.0736 (12)	
H5	0.4662	−0.6269	−0.1438	0.088*	
C6	0.5487 (3)	−0.5386 (5)	−0.18431 (16)	0.0840 (14)	
H6	0.5723	−0.6246	−0.1927	0.101*	
C7	0.5774 (3)	−0.4129 (5)	−0.20176 (15)	0.0766 (12)	
H7	0.6178	−0.4141	−0.2231	0.092*	
C8	0.5453 (2)	−0.2859 (4)	−0.18817 (13)	0.0613 (9)	
H8	0.5657	−0.2006	−0.1994	0.074*	
C9	0.2622 (2)	−0.2733 (3)	−0.17542 (11)	0.0441 (7)	
C10	0.2728 (2)	−0.1978 (4)	−0.21777 (12)	0.0551 (9)	
H10	0.3176	−0.1322	−0.2164	0.066*	
C11	0.2169 (3)	−0.2193 (4)	−0.26239 (14)	0.0650 (10)	
H11	0.2250	−0.1686	−0.2906	0.078*	
C12	0.1505 (3)	−0.3137 (4)	−0.26511 (14)	0.0653 (10)	
H12	0.1139	−0.3288	−0.2951	0.078*	
C13	0.1381 (2)	−0.3865 (3)	−0.22331 (15)	0.0646 (10)	
H13	0.0915	−0.4485	−0.2247	0.078*	
C14	0.1942 (2)	−0.3683 (3)	−0.17905 (13)	0.0565 (9)	
H14	0.1861	−0.4209	−0.1512	0.068*	
C15	0.3547 (2)	0.0010 (3)	−0.10271 (10)	0.0444 (7)	
C16	0.2748 (2)	0.0582 (4)	−0.11998 (12)	0.0605 (9)	
H16	0.2388	0.0144	−0.1462	0.073*	
C17	0.2477 (3)	0.1802 (4)	−0.09865 (14)	0.0684 (11)	
H17	0.1940	0.2192	−0.1107	0.082*	
C18	0.3005 (3)	0.2443 (3)	−0.05941 (14)	0.0602 (10)	
H18	0.2825	0.3262	−0.0447	0.072*	
C19	0.3790 (2)	0.1866 (3)	−0.04242 (12)	0.0519 (8)	
C20	0.4081 (2)	0.0654 (3)	−0.06351 (11)	0.0498 (8)	
H20	0.4624	0.0280	−0.0516	0.060*	
N1	0.37987 (17)	−0.1309 (2)	−0.12306 (9)	0.0436 (6)	
O1	0.49900 (16)	−0.0328 (3)	−0.14536 (9)	0.0642 (7)	
S1	0.37419 (7)	−0.41236 (9)	−0.10170 (3)	0.0632 (3)	
C21	0.3612 (7)	−0.228 (2)	0.0506 (4)	0.146 (15)	0.5
H21A	0.4040	−0.2957	0.0440	0.219*	0.5
H21B	0.3479	−0.2455	0.0832	0.219*	0.5
H21C	0.3831	−0.1330	0.0489	0.219*	0.5
C22	0.2809 (6)	−0.2458 (12)	0.0125 (3)	0.135 (7)	0.5
C23	0.2341 (8)	−0.1264 (10)	−0.0081 (4)	0.123 (8)	0.5
H23	0.2537	−0.0351	0.0015	0.147*	0.5
C24	0.1585 (7)	−0.1422 (11)	−0.0426 (4)	0.145 (9)	0.5
H24	0.1283	−0.0618	−0.0558	0.174*	0.5
C25	0.1282 (5)	−0.2785 (13)	−0.0573 (3)	0.160 (14)	0.5
H25	0.0779	−0.2893	−0.0803	0.192*	0.5
C26	0.1743 (6)	−0.3985 (10)	−0.0374 (3)	0.086 (3)	0.5
H26	0.1546	−0.4896	−0.0470	0.103*	0.5
C27	0.2498 (6)	−0.3820 (10)	−0.0028 (3)	0.105 (6)	0.5
H27	0.2800	−0.4626	0.0102	0.125*	0.5

### Atomic displacement parameters (Å^2^)

**Table:**

	*U*^11^	*U*^22^	*U*^33^	*U*^12^	*U*^13^	*U*^23^
Br1	0.0933 (4)	0.0782 (3)	0.0831 (3)	0.0078 (2)	−0.0077 (2)	−0.0405 (2)
C1	0.054 (2)	0.0439 (17)	0.0465 (18)	−0.0039 (14)	0.0166 (15)	−0.0027 (13)
C2	0.055 (2)	0.0477 (18)	0.0418 (17)	0.0015 (16)	0.0104 (15)	−0.0051 (13)
C3	0.050 (2)	0.0543 (18)	0.0451 (18)	0.0068 (15)	−0.0007 (15)	−0.0095 (14)
C4	0.058 (2)	0.0494 (18)	0.0483 (18)	0.0078 (16)	−0.0085 (15)	−0.0059 (14)
C5	0.077 (3)	0.050 (2)	0.083 (3)	0.0155 (19)	−0.017 (2)	−0.0109 (18)
C6	0.076 (3)	0.082 (3)	0.086 (3)	0.043 (3)	−0.010 (2)	−0.031 (2)
C7	0.063 (3)	0.099 (3)	0.064 (2)	0.026 (2)	−0.0003 (19)	−0.021 (2)
C8	0.050 (2)	0.080 (2)	0.053 (2)	0.0138 (19)	0.0033 (17)	−0.0137 (17)
C9	0.050 (2)	0.0359 (14)	0.0486 (18)	0.0026 (14)	0.0144 (15)	−0.0056 (12)
C10	0.056 (2)	0.0550 (19)	0.055 (2)	−0.0074 (16)	0.0098 (17)	−0.0003 (15)
C11	0.070 (3)	0.070 (2)	0.054 (2)	0.003 (2)	0.0094 (19)	−0.0010 (17)
C12	0.067 (3)	0.056 (2)	0.067 (2)	0.009 (2)	−0.0057 (19)	−0.0172 (18)
C13	0.056 (3)	0.0447 (18)	0.091 (3)	−0.0053 (17)	0.004 (2)	−0.0168 (19)
C14	0.058 (2)	0.0423 (17)	0.070 (2)	−0.0031 (16)	0.0157 (18)	−0.0022 (15)
C15	0.056 (2)	0.0394 (15)	0.0404 (16)	−0.0004 (14)	0.0144 (15)	−0.0006 (12)
C16	0.062 (2)	0.062 (2)	0.054 (2)	0.0100 (18)	−0.0033 (17)	−0.0125 (16)
C17	0.065 (3)	0.066 (2)	0.069 (2)	0.024 (2)	−0.0027 (19)	−0.0134 (19)
C18	0.075 (3)	0.0435 (18)	0.064 (2)	0.0102 (17)	0.015 (2)	−0.0054 (15)
C19	0.063 (2)	0.0442 (16)	0.0478 (18)	0.0006 (16)	0.0085 (16)	−0.0081 (14)
C20	0.054 (2)	0.0458 (17)	0.0496 (18)	0.0016 (15)	0.0098 (15)	−0.0043 (14)
N1	0.0467 (17)	0.0403 (13)	0.0457 (14)	−0.0042 (11)	0.0132 (12)	−0.0079 (11)
O1	0.0639 (17)	0.0552 (14)	0.0797 (16)	−0.0133 (12)	0.0304 (13)	−0.0105 (12)
S1	0.0802 (7)	0.0477 (5)	0.0597 (5)	−0.0038 (4)	0.0061 (5)	0.0101 (4)
C21	0.062 (11)	0.31 (5)	0.074 (10)	−0.006 (17)	0.029 (9)	0.029 (15)
C22	0.131 (17)	0.196 (15)	0.104 (13)	−0.034 (15)	0.096 (13)	−0.052 (12)
C23	0.147 (16)	0.109 (14)	0.143 (13)	−0.016 (13)	0.118 (12)	−0.049 (13)
C24	0.19 (2)	0.097 (10)	0.18 (2)	0.028 (11)	0.128 (17)	0.041 (11)
C25	0.120 (17)	0.26 (4)	0.123 (15)	−0.085 (18)	0.076 (12)	−0.080 (18)
C26	0.088 (8)	0.094 (7)	0.084 (7)	0.004 (6)	0.040 (6)	0.014 (6)
C27	0.174 (18)	0.056 (8)	0.106 (10)	0.002 (9)	0.088 (11)	0.002 (6)

### Geometric parameters (Å, º)

**Table:**

Br1—C19	1.895 (3)	C13—C14	1.383 (5)
C1—H1	0.9800	C14—H14	0.9300
C1—C9	1.513 (5)	C15—C16	1.376 (4)
C1—N1	1.464 (4)	C15—C20	1.382 (4)
C1—S1	1.815 (3)	C15—N1	1.435 (4)
C2—C3	1.494 (4)	C16—H16	0.9300
C2—N1	1.362 (4)	C16—C17	1.380 (5)
C2—O1	1.227 (4)	C17—H17	0.9300
C3—C4	1.396 (5)	C17—C18	1.377 (5)
C3—C8	1.404 (5)	C18—H18	0.9300
C4—C5	1.398 (5)	C18—C19	1.357 (5)
C4—S1	1.750 (4)	C19—C20	1.384 (4)
C5—H5	0.9300	C20—H20	0.9300
C5—C6	1.379 (6)	C21—H21A	0.9600
C6—H6	0.9300	C21—H21B	0.9600
C6—C7	1.372 (6)	C21—H21C	0.9600
C7—H7	0.9297	C21—C22	1.5069
C7—C8	1.365 (5)	C22—C23	1.4022
C8—H8	0.9300	C22—C27	1.4024
C9—C10	1.387 (5)	C23—H23	0.9300
C9—C14	1.382 (4)	C23—C24	1.3963
C10—H10	0.9300	C24—H24	0.9300
C10—C11	1.392 (5)	C24—C25	1.3964
C11—H11	0.9300	C25—H25	0.9300
C11—C12	1.361 (5)	C25—C26	1.3966
C12—H12	0.9300	C26—H26	0.9300
C12—C13	1.369 (5)	C26—C27	1.3963
C13—H13	0.9300	C27—H27	0.9300
			
C9—C1—H1	105.9	C16—C15—N1	119.8 (3)
C9—C1—S1	112.2 (2)	C20—C15—N1	120.1 (3)
N1—C1—H1	105.9	C15—C16—H16	119.8
N1—C1—C9	115.8 (2)	C15—C16—C17	120.4 (3)
N1—C1—S1	110.4 (2)	C17—C16—H16	119.8
S1—C1—H1	105.9	C16—C17—H17	120.0
N1—C2—C3	117.7 (3)	C18—C17—C16	119.9 (3)
O1—C2—C3	120.8 (3)	C18—C17—H17	120.0
O1—C2—N1	121.5 (3)	C17—C18—H18	120.4
C4—C3—C2	123.5 (3)	C19—C18—C17	119.3 (3)
C4—C3—C8	119.3 (3)	C19—C18—H18	120.4
C8—C3—C2	117.1 (3)	C18—C19—Br1	119.0 (2)
C3—C4—C5	119.4 (4)	C18—C19—C20	122.0 (3)
C3—C4—S1	120.8 (2)	C20—C19—Br1	119.0 (3)
C5—C4—S1	119.7 (3)	C15—C20—C19	118.5 (3)
C4—C5—H5	120.3	C15—C20—H20	120.8
C6—C5—C4	119.4 (4)	C19—C20—H20	120.8
C6—C5—H5	120.3	C2—N1—C1	122.8 (2)
C5—C6—H6	119.2	C2—N1—C15	120.2 (2)
C7—C6—C5	121.6 (4)	C15—N1—C1	116.9 (2)
C7—C6—H6	119.2	C4—S1—C1	96.77 (15)
C6—C7—H7	120.3	H21A—C21—H21B	109.5
C8—C7—C6	119.6 (4)	H21A—C21—H21C	109.5
C8—C7—H7	120.1	H21B—C21—H21C	109.5
C3—C8—H8	119.6	C22—C21—H21A	109.5
C7—C8—C3	120.7 (4)	C22—C21—H21B	109.5
C7—C8—H8	119.6	C22—C21—H21C	109.5
C10—C9—C1	122.7 (3)	C23—C22—C21	121.0
C14—C9—C1	119.4 (3)	C23—C22—C27	118.1
C14—C9—C10	117.8 (3)	C27—C22—C21	120.9
C9—C10—H10	119.7	C22—C23—H23	119.4
C9—C10—C11	120.5 (3)	C24—C23—C22	121.1
C11—C10—H10	119.7	C24—C23—H23	119.4
C10—C11—H11	119.7	C23—C24—H24	119.9
C12—C11—C10	120.6 (4)	C23—C24—C25	120.1
C12—C11—H11	119.7	C25—C24—H24	119.9
C11—C12—H12	120.3	C24—C25—H25	120.3
C11—C12—C13	119.5 (3)	C24—C25—C26	119.4
C13—C12—H12	120.3	C26—C25—H25	120.3
C12—C13—H13	119.8	C25—C26—H26	119.9
C12—C13—C14	120.4 (3)	C27—C26—C25	120.1
C14—C13—H13	119.8	C27—C26—H26	119.9
C9—C14—C13	121.1 (3)	C22—C27—H27	119.5
C9—C14—H14	119.4	C26—C27—C22	121.1
C13—C14—H14	119.4	C26—C27—H27	119.5
C16—C15—C20	119.9 (3)		
			
Br1—C19—C20—C15	179.2 (2)	C17—C18—C19—Br1	−179.5 (3)
C1—C9—C10—C11	−177.6 (3)	C17—C18—C19—C20	0.4 (6)
C1—C9—C14—C13	176.3 (3)	C18—C19—C20—C15	−0.7 (5)
C2—C3—C4—C5	−175.0 (3)	C20—C15—C16—C17	0.5 (5)
C2—C3—C4—S1	3.0 (5)	C20—C15—N1—C1	125.6 (3)
C2—C3—C8—C7	176.0 (3)	C20—C15—N1—C2	−58.2 (4)
C3—C2—N1—C1	−10.2 (4)	N1—C1—C9—C10	6.7 (4)
C3—C2—N1—C15	173.8 (3)	N1—C1—C9—C14	−170.0 (3)
C3—C4—C5—C6	0.0 (5)	N1—C1—S1—C4	−54.9 (2)
C3—C4—S1—C1	31.4 (3)	N1—C2—C3—C4	−20.5 (5)
C4—C3—C8—C7	−0.2 (5)	N1—C2—C3—C8	163.5 (3)
C4—C5—C6—C7	−1.6 (6)	N1—C15—C16—C17	176.0 (3)
C5—C4—S1—C1	−150.5 (3)	N1—C15—C20—C19	−175.2 (3)
C5—C6—C7—C8	2.3 (6)	O1—C2—C3—C4	159.5 (3)
C6—C7—C8—C3	−1.4 (6)	O1—C2—C3—C8	−16.5 (5)
C8—C3—C4—C5	0.9 (5)	O1—C2—N1—C1	169.7 (3)
C8—C3—C4—S1	179.0 (2)	O1—C2—N1—C15	−6.2 (4)
C9—C1—N1—C2	−77.8 (4)	S1—C1—C9—C10	−121.2 (3)
C9—C1—N1—C15	98.2 (3)	S1—C1—C9—C14	62.0 (4)
C9—C1—S1—C4	75.8 (3)	S1—C1—N1—C2	51.0 (3)
C9—C10—C11—C12	0.6 (5)	S1—C1—N1—C15	−132.9 (2)
C10—C9—C14—C13	−0.6 (5)	S1—C4—C5—C6	−178.1 (3)
C10—C11—C12—C13	1.0 (6)	C21—C22—C23—C24	−178.5
C11—C12—C13—C14	−2.4 (5)	C21—C22—C27—C26	178.5
C12—C13—C14—C9	2.2 (5)	C22—C23—C24—C25	−0.1
C14—C9—C10—C11	−0.8 (5)	C23—C22—C27—C26	−0.3
C15—C16—C17—C18	−0.9 (6)	C23—C24—C25—C26	−0.1
C16—C15—C20—C19	0.3 (5)	C24—C25—C26—C27	0.1
C16—C15—N1—C1	−49.8 (4)	C25—C26—C27—C22	0.1
C16—C15—N1—C2	126.4 (3)	C27—C22—C23—C24	0.3
C16—C17—C18—C19	0.4 (6)		

### Hydrogen-bond geometry (Å, º)

Cg(X) = center of gravity of ring (X); D—H···Cg(X) = angle of the D—H bond with the π plane

**Table:**

*D*—H···*A*	*D*—H	H···*A*	*D*···*A*	*D*—H···*A*
C1—H1···*Cg*5	0.98	2.66	3.5802 (6)	156
C6—H6···*Cg*3^i^	0.93	2.83	3.6823 (6)	153

Symmetry code: (i) −*x*−1, −*y*, −*z*.
